# Assessing the causal and independent impact of parity-related reproductive factors on risk of breast cancer subtypes

**DOI:** 10.1186/s12916-025-04375-6

**Published:** 2025-10-01

**Authors:** Claire Prince, Laura D. Howe, Eleanor Sanderson, Gemma C. Sharp, Abigail Fraser, Bethan Lloyd-Lewis, Rebecca C. Richmond

**Affiliations:** 1 MRC Integrative Epidemiology Unit, University of Bristol, Bristol, UK; 2 Population Health Sciences, Bristol Medical School, University of Bristol, Bristol, UK; 3 School of Psychology, University of Exeter, Exeter, UK; 4 School of Cellular and Molecular Medicine, University of Bristol, Bristol, UK

**Keywords:** Reproductive factors, Breast cancer risk, Mendelian randomization, UK Biobank, BCAC

## Abstract

**Background:**

Observational evidence proposes a protective effect of having children and an early first pregnancy on breast cancer development; however, the causality of this association remains uncertain. Here, we assess whether parity-related reproductive factors impact breast cancer risk independently of each other and other causally related or genetically correlated factors: adiposity, age at menarche, and age at menopause.

**Methods:**

We used genetic data from UK Biobank for reproductive factors and adiposity, and the Breast Cancer Association Consortium for risk of overall, estrogen receptor (ER) positive and negative breast cancer, and breast cancer subtypes. We applied univariable and multivariable Mendelian randomization (MR) to estimate genetically predicted direct effects of ever parous status, ages at first birth and last birth, and number of births on breast cancer risk.

**Results:**

We found limited evidence for a genetically predicted protective effect of an earlier age at first birth on breast cancer risk. While the univariable analysis revealed later age at first birth decreased ER-negative breast cancer risk (odds ratio (OR): 0.76; 95% confidence interval: 0.61, 0.95 per standard deviation (SD) increase in age at first birth), this effect attenuated with separate adjustment for age at menarche (potential confounder) (OR: 0.83; 0.62, 1.06) and age at menopause (genetically correlated factor) (OR: 0.80; 0.66, 1.01). Furthermore, we found evidence that a later age at first birth decreased HER2-enriched breast cancer risk but only after adjusting for number of births (potential mediator) (OR: 0.28; 0.11, 0.57 per SD increase in age at first birth).

In the multivariable analysis, we found little evidence for genetically predicted effects of ever-parous status, age at last birth, or number of births on breast cancer risk; however, analyses of ever-parous status and age at last birth were limited by weak instruments.

**Conclusions:**

This study found minimal evidence of a genetically predicted protective effect of earlier age at first birth on breast cancer risk, while identifying some evidence for a genetically predicted adverse effect on ER-negative breast cancer risk. However, weak instruments limited the multivariable analysis of ever parous status and age at last birth, which may be improved with larger sample sizes.

**Supplementary Information:**

The online version contains supplementary material available at 10.1186/s12916-025-04375-6.

## Background

Reproductive factors relating to pregnancy, including ever having children; the number of children a woman has; and age at first and last birth, have been implicated in the development of breast cancer [1].

Having children has been associated with reduced breast cancer risk [2], and this is thought to be due to pregnancy stimulating mammary epithelial lobule differentiation to its mature state, reducing the susceptibility to mutation [3]. Additionally, parous, compared to nulliparous, women have lower estrogen responsiveness via a downregulation of estrogen receptor (ER)α and upregulation of ERβ [4]. There may be an increased risk of mutation and development of breast cancer among nulliparous women due to the proliferative and genotoxic nature of estrogen [5, 6].

Experiencing a first pregnancy later in life has been associated with an increased breast cancer risk [7–9], with one meta-analysis of Nordic studies identifying a risk ratio of 1.40; 95% confidence interval (CI): 1.15, 1.70, in women whose first pregnancy was at or above age 35, compared to under 20 [9]. The protective effect of an earlier first pregnancy against breast cancer risk may be due to there being a shorter interval between menarche and first pregnancy, when there is a higher number of undifferentiated mammary cells which are more susceptible to mutation-causing events [6]. In addition, experiencing the first pregnancy later in life may increase risk because proliferation of the mammary cells during pregnancy could exacerbate underlying mutations that arise during the high-risk window prior [7, 10]. There appears to be little difference in risk if the first pregnancy occurs between 30 and mid-30s compared to nulliparous women, while having the first pregnancy after the age of 35 may increase risk [7, 9]. However, evidence for this increased risk is not consistent across all studies [9, 11].

A short-term adverse effect of pregnancy on breast cancer risk has also been observed (for women aged 35 years at first birth, 5 years post-delivery odds ratio (OR): 1.26; 1.10, 1.44, compared to nulliparous women) [12]. This risk could occur due to elevated levels of ovarian hormones during pregnancy, which have proliferative effects, increasing the chance of a mutation-causing event [5]. In addition, involution, which occurs following lactation, involves programmed cell death and tissue remodelling of the breast, which has pro-oncogenic properties similar to wound healing and inflammation [6].

The long-term protective benefits of pregnancy may not emerge until at least 10 years after delivery [12], with a proposed cross-over point where the group at higher risk changes from parous women to nulliparous women [12]. Furthermore, there is some evidence that this point may be 24 years post-delivery [13].

Considering the role of multiparity, there is evidence to suggest that each additional pregnancy conveys further protection beyond an early age at first pregnancy [14]. Other studies show that a higher age at last birth is associated with higher breast cancer risk [8, 15], with one study reporting an increased breast cancer risk for every 5-year rise in age at last birth (OR: 1.09; 1.04, 1.13) [15]. However, the mechanisms by which the number of births and age at last birth lead to breast cancer are unclear [8, 16].

Observational studies are known to be limited by confounding bias as it is difficult to capture all confounders accurately. Mendelian randomization (MR), a method less likely to be affected by confounding and reverse causation [17, 18], has been used to assess the causal relationship between reproductive factors and breast cancer risk by using genetic variants robustly associated with the exposure as instruments. MR studies have consistently found little evidence of an effect of age at first birth (Vabistsevits et al. OR 0.92; 0.79, 1.07) [19, 20], but interestingly, some evidence for a protective effect of a later age at last birth on breast cancer risk (OR 0.69; 0.54, 0.88), in contrast to observational data [20]. An MR study found that an inverse relationship exists between a higher number of births and breast cancer risk, although confidence intervals for the effect estimate spanned the null (OR 0.70; 0.44, 1.11) [19].

It is currently unclear how each reproductive event affects risk in isolation since these traits are highly correlated with, and/or causally linked to, other reproductive factors as well as age at menarche and menopause, and adiposity measures [21, 22], which are established breast cancer risk factors [23, 24]. Multivariable MR (MVMR) is an extension of MR which can estimate the direct effect of multiple risk factors while accounting for genetic correlation between them [25, 26], which may be valuable for investigating the impact of correlated reproductive factors. The effects of reproductive factors on risk of different subtypes of breast cancer are not well understood [19, 27–33]. There is some consensus that a lower age at first birth and higher parity decrease risk of hormone-receptor positive breast cancer subtypes, although there is less consistency for the effects of these traits on risk of hormone-receptor negative subtypes [30–33]. As more women are choosing not to have children [34], or having children later in life [35], it is important to understand how these traits might convey different risk depending on subtype.

### Aims

We aimed to use univariable and multivariable MR methods to evaluate whether the observational evidence that an earlier age at first birth and ever having children decreases breast cancer risk is supported by causal inference methodology. Additionally, we aimed to assess the effects of age at last birth and number of children birthed on breast cancer risk.

We further aimed to investigate whether effects are independent of age at menarche and menopause, and adiposity measures as well as the other parity-related reproductive factors, and whether effects differ for ER-positive compared to ER-negative breast cancer. Furthermore, based on these findings, we aimed to assess the effects of reproductive factors on the risk of breast cancer subtypes.

## Methods

### UK Biobank

The UK Biobank study is a large population-based cohort of 502,682 individuals who were recruited at ages 37–73 years across the UK between 2006 and 2010. The study includes extensive health and lifestyle questionnaire data, physical measures, and biological samples from which genetic data has been generated. The study protocol is available online, and more details have been published elsewhere [36]. At recruitment, the participants gave informed consent to participate and be followed up. Unless stated otherwise, in UK Biobank, the factors investigated in the current study were derived from questionnaire responses at the baseline assessment.

#### Reproductive factors

The reproductive factors investigated in this study were: age at first live birth, age at last live birth, number of live births, and parous status (ever/never given birth at the time of assessment). We will refer to these factors when in groups or subgroups as primary exposures hereafter. To identify genetic variants robustly related to each of the reproductive factors, we performed a genome-wide association study (GWAS) for each reproductive factor among women in the UK Biobank. Further details can be found in Additional File 1 [37–39].

#### Additional variables

We additionally included age at menarche and menopause, and adiposity measures in childhood and adulthood. We will refer to these factors as adjustment variables when in groups or subgroups. Further details on how these variables are defined can be found in Additional File 1 [37–44].

We performed GWAS for age at menarche and menopause among women in the UK Biobank similarly to the primary exposures as stated above. We obtained female-only GWAS summary statistics for childhood and adulthood body size from Richardson et al. (2020), where they performed GWAS using a similar approach [42].

### Breast cancer association consortium

Overall (33,384 cases and 113,789 controls), ER-positive (69,501 cases and 105,974 controls), ER-negative (21,468 and 105,974 controls), HER2-enriched (718 cases and 20,815 controls), and triple-negative (2006 cases and 20,815 controls) breast cancer risk GWAS summary statistics were obtained from the Breast Cancer Association Consortium (BCAC). [45, 46] Further details can be found in Additional File 1 [45, 46].

### Genetic correlation

Genetic correlations between the primary exposures, adjustment variables, and breast cancer risk outcomes were calculated using linkage disequilibrium score regression (LDSC) and the UK Biobank, and BCAC GWAS summary statistics [47, 48]. Further details can be found in Additional File 1 [47, 49].

Prior to MR analyses, GWAS estimates for age at first birth, age at last birth, and number of births were standardized based on the standard deviation of the phenotypic exposure (mean: 0 and SD: 1). Further details on the analysis performed and functions used can be found in Additional File 1 [18, 25, 26, 38, 50–55].

### SNPs selected for MR analyses

Five SNPs were robustly associated with ever-parous status, 43 with age at first birth, and 10 with both age at last birth and number of births. SNPs associated with each primary exposure are shown in Table 1.

**Table 1:** The SNPs robustly associated with ever parous status, age at first birth, age at last birth*,* and number of births using a genome-wide significance threshold of 5 × 10^−8^ and after linkage disequilibrium clumping

Primary exposure	Robustly associated SNPs
Ever parous status	rs9862795, rs4870037, rs4870060, rs4598264, rs62065450
Age at first birth	rs11210939, rs1222766, rs12749691, rs2842711, rs6545313, rs1019615, rs359240, rs6723909, rs2570497, rs10496880, rs59976582, rs9815930, rs12513045, rs11743711, rs9296933, rs9482120, rs3757323, rs55988458, rs794375, rs113905912, rs13237149, rs2597399, rs7839443, rs7046483, rs34522021, rs11192193, rs9666824, rs2160514, rs2456973, rs7972441 rs4140762, rs16943403, rs7324673, rs5742915, rs7195242, rs3867518, rs9898399, rs1941954, rs1396650, rs71367544, rs751858, rs6079584, rs17836979
Age at last birth	rs2166172, rs2161990, rs359253, rs4438499, rs9815930, rs12485556, rs2581764, rs7467480, rs11192193, rs16943403
Number of births	rs17837951, rs35600310, rs9862795, rs4856591, rs4869737, rs4305732, rs2627197, rs174555, rs199441, rs41156

### STROBE-MR

The Strengthening the Reporting of Observational Studies in Epidemiology using Mendelian Randomization (STROBE-MR) guidelines have been followed in the analysis of this work (Additional File 1) [56, 57].

### Univariable analysis

We performed univariable MR (UVMR) of the primary exposures on overall, ER-positive, and ER-negative breast cancer risk. We additionally performed UVMR on the primary exposures that appear to have an effect on the risk of ER-negative breast cancer, on ER-negative breast cancer subtypes (HER2-enriched and triple-negative).

Mendelian randomization has three main assumptions: the relevance assumption states the genetic variants instrumented are associated with the exposure of interest; the independence assumption states the genetic variants and outcome under investigation do not share any common causes; the exclusion restriction assumption states that the instrumented genetic variants do not affect the outcomes via any pathway other than the exposure being examined, i.e., there are no pleiotropic effects [53].

To evaluate whether the genetic instruments were robustly associated with each primary exposure, we determined the mean *F*-statistic for each of these exposures [53], and considered an *F*-statistic of 10 or above as indicative of a strong instrument. To evaluate the third assumption, we performed MR using additional methods: weighted mode [58], weighted median [59], and MR Egger [60, 61], as these methods allow for directional pleiotropy. Additionally, assessing the MR Egger regression intercept was used as another way to consider the presence of directional pleiotropy for each relationship investigated [60]. We also applied another MR method to assess for directional pleiotropy: MR-PRESSO (Mendelian Randomisation Pleiotropy RESidual Sum and Outlier). This method detects and accounts for horizontal pleiotropy by correcting for any single nucleotide polymorphisms (SNPs) identified as outliers [52].

### Multivariable analysis

Given the evidence for genetic correlation and causal relationship between the primary exposures and additional variables, we performed MVMR to evaluate the direct causal effect of the primary exposures on overall, ER-positive, and ER-negative breast cancer risk. We additionally performed MVMR of the primary exposures that appeared to have an effect on risk of ER-negative breast cancer, on breast cancer subtypes (HER2-enriched and triple-negative).

**Fig. 1:**
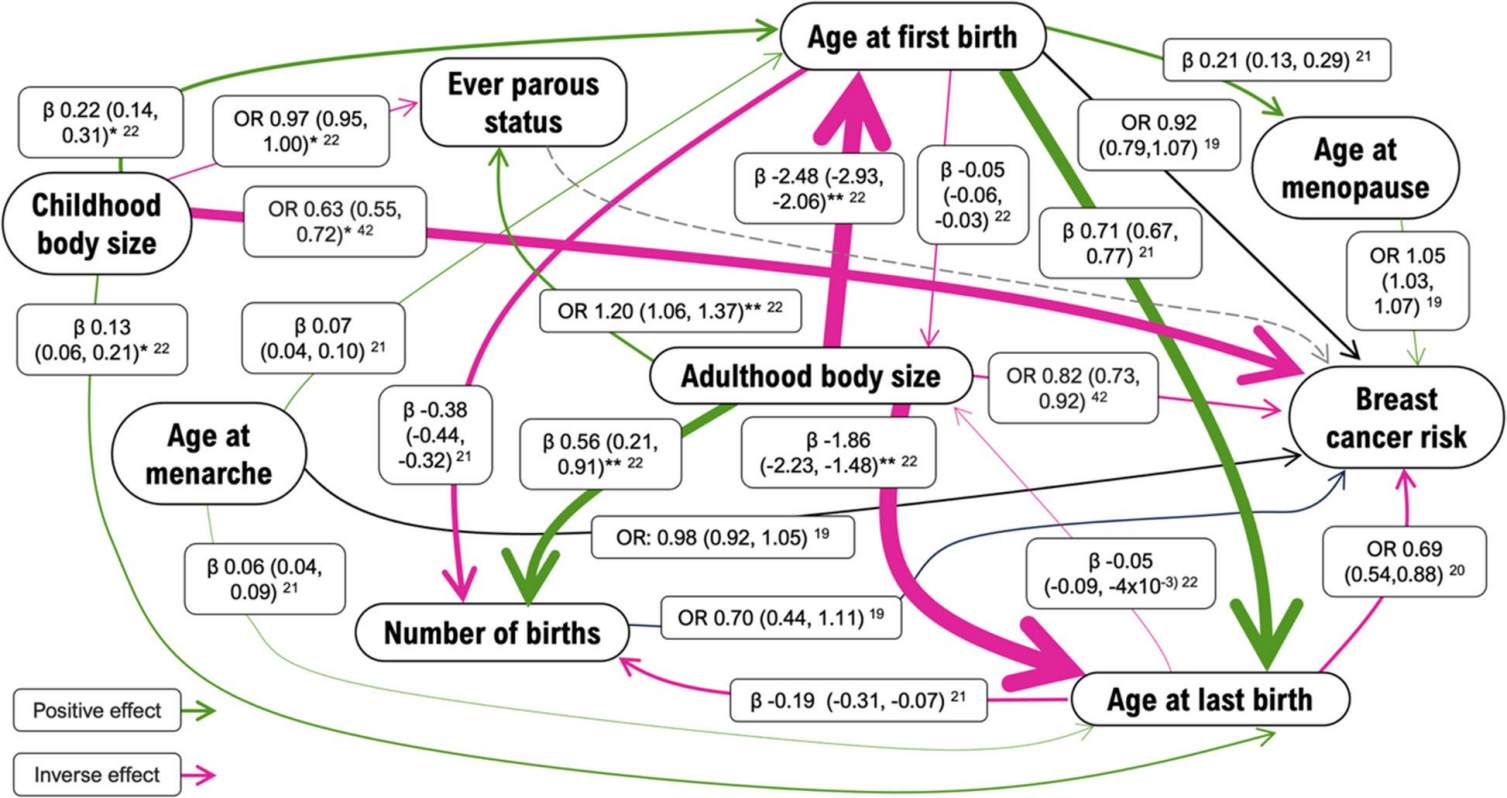
Causal relationships estimated using Mendelian randomization in previous work (Vabistsevits et al. (2022) [19], Prince et al. (2022) [21], Prince et al. (2023) [22], Richardson et al. (2020) [42]). OR, odds ratio; 95% confidence interval. The width of the line/arrowhead indicates the magnitude of the estimate. Given ever parous status and breast cancer risk are binary traits, odds ratios are presented where this trait is an outcome. Estimates are univariable unless indicated otherwise. * indicates multivariable analyses adjusted for adulthood body size. ** indicates multivariable analysis adjusted for childhood body size

Previous research has identified genetic correlation and causal relationships between the primary exposures, other established and potential breast cancer risk factors, and breast cancer risk. The causal relationships are demonstrated in Fig. 1, represented by univariable Mendelian randomization estimates from this previous work. Based on this evidence, the factors that were considered for MVMR adjustment were (1) breast cancer risk factors identified to have a causal effect on the exposure and thus a potential confounder, (2) risk factors identified to have a causal effect from the exposure and thus a potential confounder, and (3) traits that we have shown have a genetic correlation with the exposure but for which there is little evidence for a causal relationship with the exposure [21, 22]. It is important to adjust for genetically correlated risk factors to isolate the effect of the exposure and avoid confounding due to shared genetic architecture [25].

**Fig. 2:**
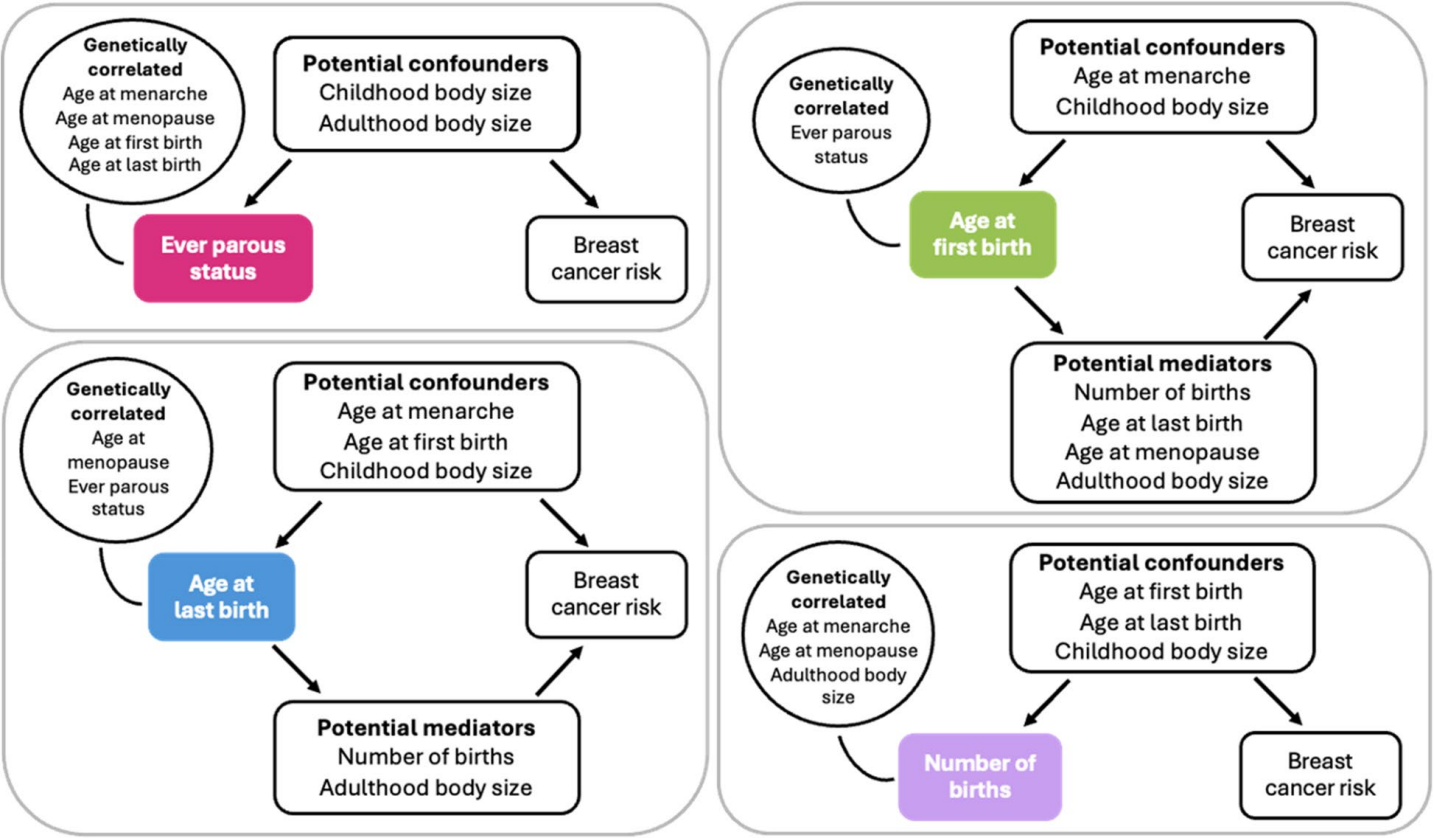
Variables included in multivariable Mendelian randomization analyses

We adjusted for the adjustment variables shown in Fig. 2 in turn in relation to each breast cancer risk outcome.

It is worth noting that the ever-parous status phenotype was derived from the number of births, and therefore the two variables were not included in a multivariable model together.

It may seem implausible to adjust for ever parous status in the analysis of age at first and last birth on breast cancer risk since these events only occur in women who have given birth and therefore ever parous status could not observationally confound or mediate this relationship. However, a recent study has shown that performing MR without adjusting for a factor that was conditioned on in one of the exposure or outcome GWAS can bias the resulting MR estimate, and adjusting for that factor can allow estimation of direct effects [62]. While age at first and last birth are conditioned on ever parous status since the GWAS was for these traits only being performed among parous women, the GWAS of breast cancer risk was not performed solely on parous women or adjusted for ever parous status, and so it is important to adjust for ever parous status in a multivariable model.

We evaluated the instrument strength in the multivariable analysis using a conditional *F*-statistic [63], and used a modified form of Cochran’s *Q*-statistic to evaluate evidence of horizontal pleiotropy [54]. As we identified weak instruments and/or evidence of pleiotropy across the multivariable analyses, we performed MVMR estimation using *Q*-statistic minimization and present this as the primary analysis. This method has been developed to be robust to weak instruments and balanced heterogeneity, and has been described extensively elsewhere [54].

We did not use this method when the *F*-statistic falls below 4, since the method does not perform well in this circumstance [54]. Additionally, given MVMR estimation using *Q*-statistic minimization is only robust to balanced pleiotropy, we performed MVMR using the MR Egger method to evaluate the presence of directional pleiotropy, which would violate the exclusion restriction assumption [55, 64]. This analysis is performed similarly to the initial MVMR analysis in relation to overall, ER-positive, and ER-negative breast cancer risk.

## Results

### UK Biobank

Two hundred seventy-three thousand two hundred thirty-eight women from UK Biobank were included. The mean age at assessment was 56 years (SD = 8); further sample characteristics are shown in Table 2.

**Table 2:** UK Biobank study characteristics included in this study

Trait		*N*	Mean (SD)
Age at menarche (years)		243,898	13.0 (1.6)
Age at first live birth (years)		203,606	25.9 (5.1)
Age at last live birth (years)		203,356	30.1 (5.2)
Age at menopause (years)		143,791	49.7 (5.1)
Number of live births		250,746	1.8 (1.2)
Adulthood BMI		250,746	27.1 (5.2)
**Trait**		**% (*N*)**	
Never parous		18.69 (49 358)	
Childhood body size	About average	50.47 (135 399)	
	Thinner	31.80 (85 316)	
	Plumper	17.74 (47 585)	

*N* Sample size, *SD* Standard deviation, *IQR* Interquartile range. Childhood body size is defined as a comparative body size measure where participants were asked, “When you were 10 years old, compared to average would you describe yourself as:” and were given the options: “Thinner,” “Plumper,” and “About average”

#### Genome-wide association studies

Table 3 displays the number of SNPs associated with each primary exposure at genome-wide significance (*p* value < 5 × 10^−8^) after linkage disequilibrium clumping and harmonizing with the outcome.

**Table 3:** Univariable instrument strength of each trait of interest

Trait	Trait *N*	Breast cancer risk	nSNPs	*F*-statistic
Age at first live birth	203,606	Overall	34	39.18
		ER status	41	38.28
		Subtypes	34	39.18
Age at last live birth	203,356	Overall	8	34.11
		ER status	9	33.98
		Subtypes	8	34.11
Number of live births	250,746	Overall	8	43.64
		ER status	9	42.79
		Subtypes	8	43.64
Ever parous status	250,746	Overall	4	39.43
		ER status	4	39.43
		Subtypes	4	39.43

*N* Sample size, *nSNPs* Number of SNPs

#### Genetic correlation

**Fig. 3:**
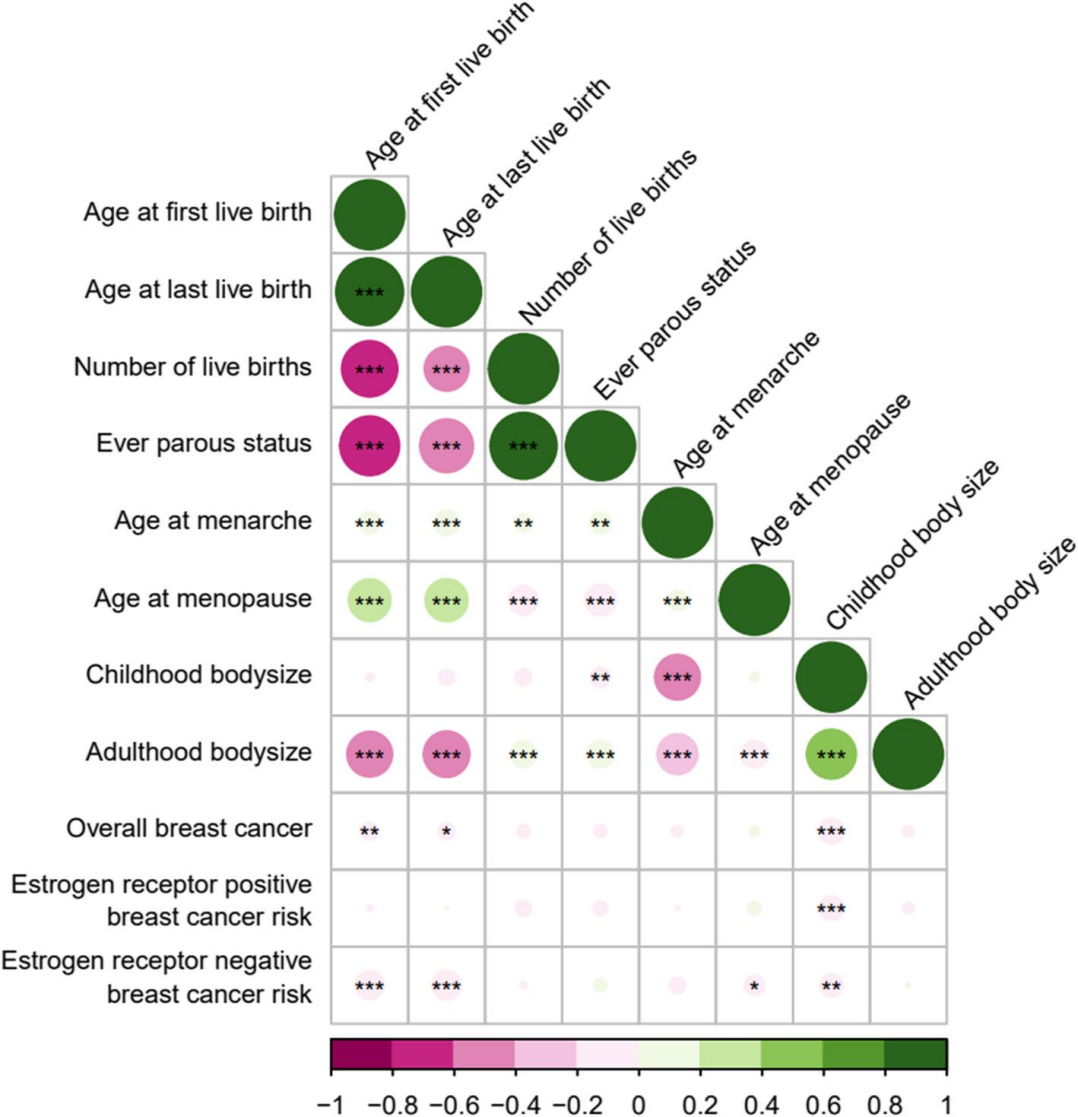
Genetic correlation between primary exposures, adjustment variables, and breast cancer risk. * *p*-value < 0.05, ** *p*-value < 0.01, *** *p*-value < 0.001

Many of the primary exposures and adjustment variables were strongly genetically correlated. Exceptions were between childhood body size and age at first birth, age at last birth and number of births (Fig. 3, Additional File 2: Table 1). In addition, age at first and last birth were inversely genetically correlated with overall and ER-negative breast cancer risk but not ER-positive. Ever parous status and number of births were not genetically correlated with any of the breast cancer risk outcomes (Fig. 3, Additional File 2: Table 1).

### Mendelian randomization

In the univariable MR analysis, primary exposures had an *F*-statistic over the standard threshold of 10 (Table 3). However, in the multivariable analysis, the *F*-statistic fell below this threshold for all the primary exposures, with adjustment for at least one factor in the MVMR analysis (Additional File 2: Table 2).

We therefore present MVMR estimated using *Q*-statistic minimization, which has been developed to be robust to weak instruments and balanced heterogeneity as the primary analysis.

However, where the instruments were deemed too weak, with an *F*-statistic of less than 4, we present MVMR using the IVW method. Where this was the case is shown in Additional File 2: Table 2.

In the MVMR analysis, we adjusted for each factor in turn; thus, there were only two exposures: the primary exposure and one adjustment variable, and the outcome in each MVMR model. The evidence for genetic correlation and causal relationships between the primary exposures and adjustment variables shown above was used to inform the adjustment variable used in the multivariable models for each primary exposure.

ORs for age at first birth, age at last birth and number of births are shown as per SD in the phenotypic exposure.

#### Ever-parous status

**Fig. 4:**
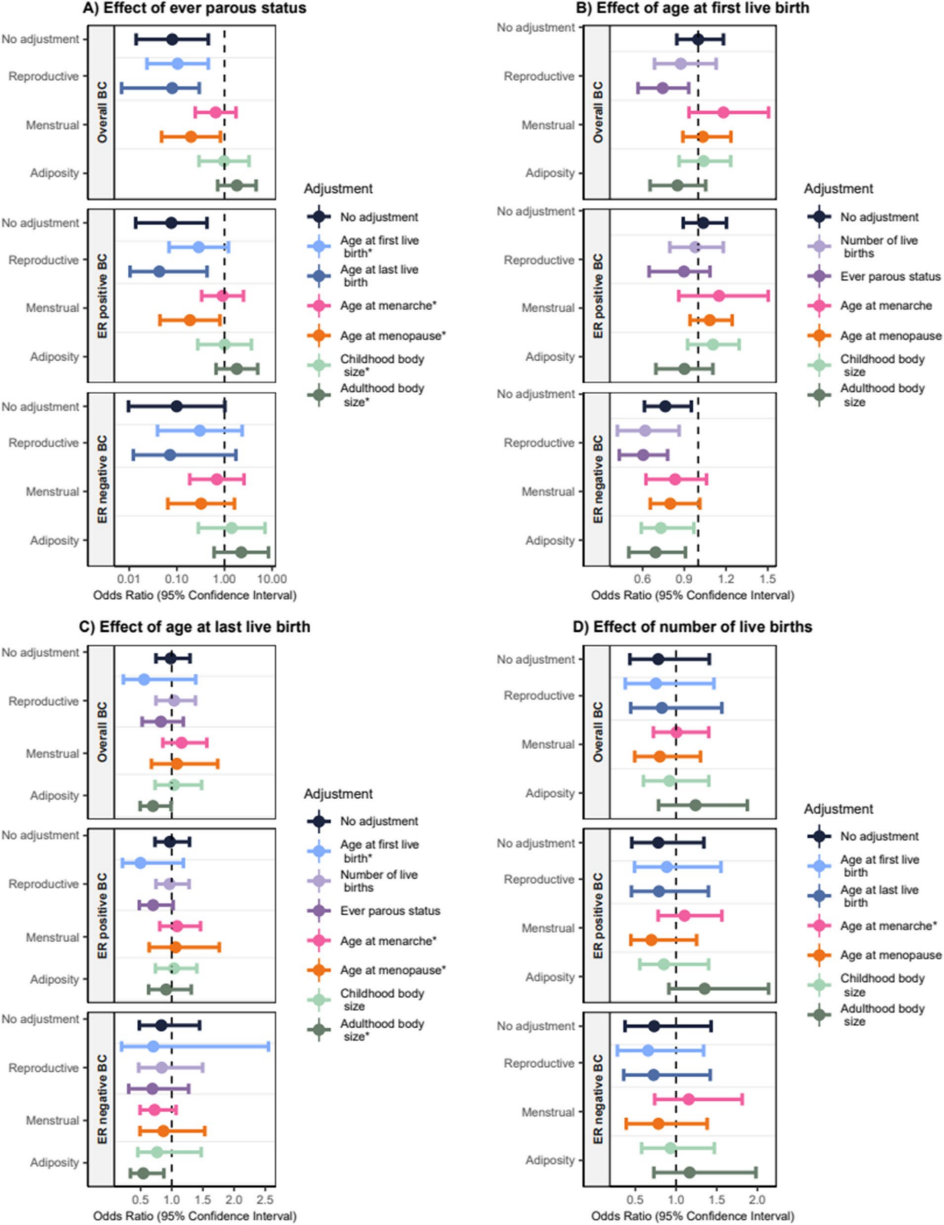
Univariable and multivariable Mendelian randomization assessing the effects of **A** ever parous status, **B** age at first birth, **C** age at last birth, and **D** number of births on overall, ER-positive, and ER-negative breast cancer risk. No adjustment indicated findings from the univariable analysis. Effect of ever parous status is presented per log odds of ever parous status, and effects of age at first birth, age at last birth, and number of births are presented per standard deviation increase in the phenotypic exposure. * Initial MVMR results are presented due to weak instruments preventing the use of MVMR estimated using *Q*-statistic minimization

Ever parous status is a binary exposure and therefore, to facilitate interpretation, the effects in Additional File 2: Table 3 and Fig. 4A have been converted from “ORs per log odds” in the main text to per doubling in the genetic liability to ever being parous.

In the univariable analysis, we identified a similar protective effect of ever parous status on overall (UVMR: 0.17; 0.05, 0.58), ER-positive breast cancer risk, and ER-negative breast cancer risk. In the multivariable analysis, the conditional *F*-statistics for ever parous status fell below 4 for all adjustments except age at last birth, and the number of SNPs used as instruments was 4 or fewer (Additional File 2: Table 2) Adjusting for age at last birth (genetically correlated factor) revealed a largely similar effect compared to the univariable analysis on overall (MVMR estimated using *Q*-statistic minimization OR: 0.17; 0.07, 0.43), ER-positive, and ER-negative breast cancer risk (Fig. 4A, Additional File 2: Table 3).

#### Age at first birth

We identified little evidence for an effect of age at first birth on overall breast cancer risk (UVMR OR: 1.00, 95% CI 0.85, 1.18); however, adjusting for ever parous status (genetically correlated factor) revealed an inverse effect (MVMR estimated using *Q*-statistic minimization OR: 0.74; 0.57, 0.93). Little evidence was identified for an effect of age at first birth on ER-positive breast cancer risk in the univariable analysis (UVMR OR: 1.04; 0.89, 1.20), with a similar effect seen across the multivariable analyses. Adjusting for age at menarche (potential confounder) revealed the largest positive effect although confidence intervals are wide, overlapping the univariable estimate and the null (MVMR estimated using *Q*-statistic minimization OR: 1.15; 0.86, 1.50). We identified an inverse effect on ER-negative breast cancer risk in the univariable analyses (UVMR OR: 0.76; 0.61, 0.95); however, this attenuated after adjustment for age at menarche (potential confounder) (MVMR estimated using *Q*-statistic minimization OR: 0.83; 0.62, 1.06) and age at menopause (potential mediator) (MVMR estimated using *Q*-statistic minimization OR: 0.80; 0.66, 1.01) (Fig. 4B, Additional File 2: Table 4).

#### Age at last birth

We found little evidence for an effect of age at last birth on overall (UVMR OR: 0.98; 0.75, 1.29), ER-positive (UVMR OR: 0.97; 0.73, 1.29) and ER-negative (UVMR OR: 0.83; 0.48, 1.45) breast cancer risk in the univariable analysis (Fig. 4C, Additional File 2: Table 5).

However, we found evidence of an inverse effect of age at last birth adjusting for adulthood body size (potential mediator) on overall (MVMR OR: 0.70; 0.49, 0.99), and ER-negative breast cancer risk (MVMR OR: 0.54; 0.34, 0.87). Stronger evidence for an effect was revealed after adjustment for adulthood body size (potential mediator) in the multivariable analysis of ER-negative breast cancer risk, although instruments were weak (Fig. 4C, Additional File 2: Table 5).

#### Number of births

We found limited evidence for an effect of number of births on overall (UVMR OR: 0.78; 0.43, 1.41), ER-positive (UVMR OR: 0.78; 0.46, 1.34) or ER-negative breast cancer risk (UVMR OR: 0.73; 0.37, 1.43) in the univariable analysis, with a similar result in the multivariable analyses (Fig. 4D, Additional File 2: Table 6).

#### Estrogen receptor negative subtypes

Since we found some evidence for an effect of age at first birth on ER-negative breast cancer risk with relatively strong instruments, we investigated the effects of age at first birth on HER2-enriched and triple-negative breast cancer risk in a similar manner as in relation to overall, ER-positive, and ER-negative breast cancer risk.

The univariable analysis revealed limited evidence of an effect of age at first birth on HER2-enriched breast cancer risk (UVMR OR: 0.78; 0.47, 1.30), while adjusting for number of births (potential mediator) (MVMR estimated using *Q*-statistic minimization OR: 0.28; 0.11, 0.57) and ever parous status (genetically correlated factor) (MVMR estimated using *Q*-statistic minimization OR: 0.38; 0.17,0.80) revealed inverse effects (Additional File 2: Table 4, Additional File 3: Fig. S1). We found minimal evidence for an effect of age at first birth on triple-negative breast cancer risk in the UVMR analysis (UVMR OR: 0.88; 0.63, 1.24) which was similar in the MVMR analysis (Additional File 2: Table 4, Additional File 3: Fig. S1).

#### Evaluating Mendelian randomization assumptions

While there was some consistency between the univariable analysis using the IVW method and the additional MR methods (MR Egger, weighted median and weighted mode), there were inconsistencies in the analysis of ever parous status and age at first birth, which may suggest pleiotropy in these analyses (Additional File 2: Table 7). However, while the MR-PRESSO methods identified some pleiotropic outliers in the univariable analysis, correcting for identified outliers did not appear to change the evidence for effects identified in the initial analysis using the IVW method (Additional File 2: Table 8). The Egger intercept test did not identify evidence for pleiotropy across any relationships investigated (Additional File 2: Table 9).

Further details on these analyses are found in Additional File 1.

In the multivariable analysis, we identified evidence of heterogeneity with some exceptions (Additional File 1, Additional File 2: Table 10). We performed the multivariable analysis using the MR Egger method to assess for pleiotropy, and the estimates were mostly consistent with the main analysis, although in many cases, the confidence intervals spanned the null. However, there were inconsistencies, suggestive of pleiotropy. The MR Egger method in the multivariable analysis revealed an inverse effect of number of births, adjusted for age at first birth, on overall (OR: 0.49; 0.26, 0.91) and ER-negative breast cancer risk (OR: 0.42; 0.19, 0.91) which was not identified in the main analysis (Additional File 2: Table 11, Additional File 3: Fig. S2). Further details on these analyses are found in Additional File 1, Additional File 3: Fig. S3–S5.

Where we identified an effect of age at first birth on HER2-enriched breast cancer risk in the multivariable IVW analysis, wide confidence intervals mostly included the null using the MR Egger method. We additionally identified minimal evidence for an effect of age at first birth on triple-negative breast cancer risk in the MVMR analysis using the MR Egger method, similarly to the IVW method (Additional File 2: Table 11).

## Discussion

In this study, we used Mendelian randomization methods to investigate the role of parity-related reproductive factors on breast cancer risk.

While univariable analysis revealed evidence to support that having children has a protective effect on breast cancer risk, we could not determine the direct effect independently of other reproductive factors and adiposity measures due to the analysis being subject to weak instruments across the multivariable models. It may be that a larger GWAS of ever parous status is required to identify a higher number of robustly associated SNPs and increase instrument strength.

Similar to previous studies, we found little evidence to support a protective effect of an earlier age at first birth on breast cancer risk in our univariable analysis [19, 20], with no evidence of a divergence towards a protective effect in the multivariable analysis. One explanation may be that the observational literature is biased by residual confounding that our study is able to avoid using MR. However, another explanation is that our study is limited by biases, such as residual pleiotropy, that have driven our MR estimates towards the null, discussed in more detail below.

We did, however, identify that an earlier age at first birth leads to increased overall breast cancer risk after adjusting for ever parous status (genetically correlated factor), and in the ER status stratified analyses, we also find evidence of this effect on ER-negative breast cancer risk, but not in relation to ER-positive breast cancer risk. This inverse effect of later age at first birth on ER-negative breast cancer risk attenuated somewhat with adjustment for age at menarche (potential confounder) and menopause (potential mediator), where confidence intervals crossed the null. While age at menarche might be a true confounder of this relationship and age at menopause may be a true mediator, the attenuation of this effect in both multivariable MR models may also be explained by shared genetic confounding. This is particularly likely in the case of age at menopause, given the positive indirect effect that may occur via age at menopause highlighted in Fig. 1, and the genetic correlation between both age at menarche and menopause, and age at first birth (Fig. 3).

When evaluating the HER2-enriched and triple-negative subtypes, we found that, after adjustment for number of births (potential mediator) and ever parous status (genetically correlated factor), an earlier age at first birth increases HER2-enriched but not triple-negative breast cancer risk.

Since ER-negative breast cancer is typically seen in premenopausal women, increased risk from an earlier first pregnancy is more likely to be related to ER-negative breast cancer. The effect of age at first birth on HER2-enriched breast cancer risk that we find could be due to an increased risk of HER2 mutations during pregnancy, leading to HER2 overexpression, driving the development of breast cancer [65]. Additionally, pregnancy is thought to increase breast cancer risk in the shorter-term because of the inflammatory and wound healing properties of mammary involution, which occurs after lactation [6].

Previous observational research has identified that having an age at first birth at or younger than 25 is associated with a lower level of estrogen post-menopause compared to nulliparous women [66]. Therefore, it would be valuable for future studies to evaluate whether levels of ovarian hormones play a role in the relationship between parity-related factors and breast cancer risk, for example, in a mediation MVMR framework.

Point estimates identified in the univariable analysis of age at last birth, and across the multivariable models suggest an adverse effect of a younger age at last birth on ER-negative breast cancer risk, which is consistent with a previous univariable MR study [20], but conflicts with the observational literature, which suggests a higher age at last birth is associated with higher breast cancer risk [8, 15].

Although it may seem implausible to adjust for ever parous status in the analysis of age at first and last birth on breast cancer risk, we highlight the importance of this adjustment in the methods section to avoid bias as described by Gilbody et al. [62]. Moreover, in the analysis of age at first birth on overall breast cancer risk, adjusting for ever parous status did modify the estimate to reveal an inverse effect, compared to the limited evidence identified in the univariable analyses. It would have been informative to perform the analysis of age at first and last birth using GWAS from the BCAC consortium performed solely in parous women. However, to our knowledge, these data are not publicly available.

Due to weak instruments, we cannot make any definitive conclusions regarding age at last birth or ever parous status for most multivariable models. Where instruments were at least slightly stronger, with an *F*-statistic of 4 or higher, in the multivariable models, we performed MVMR estimation using *Q*-statistic minimization, and the direction and strength of evidence were largely similar to the univariable model. It may be that we are capturing the effect of age at first birth since these factors are highly genetically correlated. Indeed, in the multivariable analysis of age at last birth on breast cancer risk outcomes adjusted for age at first birth, confidence intervals were particularly wide, compared to the other models with age at last birth as the exposure.

We find limited evidence that the number of births has an impact on breast cancer risk, which is consistent with a previous MR study [19].

We used the MR Egger method to assess for evidence of pleiotropy; for the most part, this method revealed similar evidence to the main analysis. In addition, using the MR Egger method, adjusting for age at first birth uncovered evidence that a higher number of children leads to reduced overall and ER-negative breast cancer risk. While this suggests the main analysis may be biased by pleiotropy, the instrument strength was deemed weak in the multivariable analyses, which limited confidence in these results and prevented further assessment of pleiotropy using robust methods [67].

### Strengths

The main strength of this study is the use of a multivariable approach allowing the evaluation of direct effects of reproductive factors on breast cancer risk. This has been informed by established genetic correlations and causal relationships between the reproductive factors of interest and other reproductive factors and adiposity measures [21, 22].

Additionally, this study uses the UK Biobank study, which has phenotypic and genetic data on a large number of individuals, which increases the likelihood of identifying strong genetic instruments for each exposure of interest. Another strength is the use of GWAS data from the BCAC consortium, allowing the investigation of the risk of breast cancer by ER status and the risk of breast cancer subtypes.

### Limitations

This study has a number of limitations that should be considered when interpreting results. Firstly, weak instruments were a key limitation in the analysis of age at last birth and ever parous status. This can be attributed to the genetic correlation that is present between these traits and the other factors included in the multivariable analyses [21], alongside the low number of SNPs that arise as genome-wide significant in relation to these traits (8 and 4 for age at last birth and ever parous status, respectively). Where the instrument strength was not deemed too weak, we additionally performed MVMR estimation using *Q*-statistic minimization that is developed to be robust to weak instruments. However, this was not possible where instruments were extremely weak (*F*-statistics < 4). It would have been useful to include multiple adjustment variables within the multivariable models, although instruments were too weak to facilitate this. Larger GWAS and stronger instruments are required to extend our multivariable approach. However, it may be that, due to the aforementioned strong genetic correlation, an exceptionally large sample size would be required to perform a fully adjusted multivariable model. Nonetheless, in our approach, by adjusting for each adjustment variable in turn, we were able to determine the extent to which each variable is responsible for any divergence away from the univariable estimate.

Secondly, as previously mentioned, we may not find a protective effect of an early age at first birth on breast cancer risk because of biases due to violations of the MR assumptions. It is plausible that we may be capturing residual pleiotropic effects as a result of age at menarche confounding the relationship between age at first birth and breast cancer risk. The univariable analysis reveals little evidence for an effect of age at first birth on ER-positive breast cancer risk, while the effect in the multivariable analysis, adjusted for age at menarche, is larger in the protective direction, although confidence intervals crossed the null. Given the conditional *F*-statistics in this multivariable model (age at first birth: 5.3, age at menarche: 25.0) and that age at menarche and first birth are strongly genetically correlated, the effect in the multivariable analysis may be biased by weak instruments or from pleiotropy via another trait. Stronger instruments for age at first birth in an MVMR model adjusted for age at menarche might reveal the protective effect seen in the observational literature.

Thirdly, the reproductive factors were self-reported, which can be unreliable. However, we can be relatively confident that reports of age at first and last birth and number of births are accurate since these are significant life events that are likely to be reliably recalled. Additionally, there has typically been good replication of the genetic scores for the reproductive traits in other cohorts [68, 69, 70].

In addition, since the reproductive factors investigated in this study are bio-social, findings may not be generalizable to other non-UK populations that have different social norms. Additionally, the findings may not be generalizable to younger populations. It would have been valuable to compare the findings in the study, where the population was predominately European, to populations with other ancestries such as Asian and African. However, while Asian and African ancestry GWAS summary statistics for breast cancer risk are available, those for the four reproductive factors investigated here are not, to the best of our knowledge.

While the reproductive factors examined in this study are in part underpinned by social mechanisms, there is evidence for heritability of these traits, giving biological plausibility to the genetic variants used in this study. Mills et al. (2021) reported a heritability estimate for age at first birth of 22% (CI: 19, 25) for women born in 1965, an increase from the previous years [71]. Additionally, heritability estimates of a range between 24 and 43% have been reported for number of births between different European cohorts [72]. However, traits that are in part explained by social mechanisms are more susceptible to genetic confounding such as population stratification, dynastic effects, and assortative mating [73]. Within family MR studies could be used to remove and evaluate this confounding [73]; however, these studies are often low powered, and the data is not readily available.

Finally, the MR analysis performed assumes a constant effect of age at first and last birth, and number of births, on breast cancer risk. Future studies could use MR to evaluate the effects of an early age at first birth compared to an average and late age, and vice versa. This approach would also enable the evaluation of whether there is a cross-over point where breast cancer risk changes from parous women to nulliparous women at a certain age at first birth as previously proposed [12]. Again, further analysis would require the identification of additional and stronger genetic instruments.

## Conclusions

Using a series of Mendelian randomization analyses, we found minimal evidence for a protective effect of early age at first birth on breast cancer risk that has been previously identified, which may be due to unmeasured biases in previous observational studies. On the other hand, we identify some evidence of an adverse effect on ER-negative breast cancer risk, which supports the notion that there is an immediate increase in breast cancer risk post-pregnancy. Understanding the mechanism behind ER-negative breast cancers is important since they have worse prognosis compared with those that are ER-positive. Future studies considering non-linear relationships between age at first birth and breast cancer risk may provide additional insights.

In addition, we show the number of births that women have has little effect on breast cancer risk. While our findings for ever parous status and age at last birth do not all concur with the existing literature from non-genetic studies, this may partly be due to limitations of the multivariable Mendelian randomization approach, specifically relating to weak conditional instrument strength, which may be overcome with larger GWAS of these traits.

## Supplementary Information

Additional file 1. Supplementary methods and results, and MR STROBE checklist.

Additional file 2: Supplementary Tables. Table 1 Genetic correlation between reproductive factors using linkage disequilibrium score regression. Table 2 Instrument strength across multivariable mendelian randomization analyses. Table 3 Mendelian randomization results for the effect of ever parous status on breast cancer risk. Table 4 Mendelian randomization results for the effect of age at first birth on breast cancer risk. Table 5 Mendelian randomization results for the effect of age at last birth on breast cancer risk. Table 6 Mendelian randomization results for the effect of number of births on breast cancer risk. Table 7 Univariable mendelian randomization results for the effects of ever parous status, age at first birth, age at last birth and number of births on breast cancer risk across methods MR Egger, weighted median and weighted mode. Table 8 MR PRESSO results for the effects of ever parous status, age at first birth, age at last birth and number of births on breast cancer risk. Table 9 Egger intercept results for the effects of ever parous status, age at first birth, age at last birth and number of births on breast cancer risk. Table 10 Test for heterogeneity for the multivariable analysis of ever parous status, age at first birth, age at last birth and number of births on breast cancer risk. Table 11 Multivariable mendelian randomization results for the effects of ever parous status, age at first birth, age at last birth and number of births on breast cancer risk using the MR Egger method. Table 12 Association between SNPs robustly associated with ever parous status, and ever parous status and breast cancer risk. Table 13 Association between SNPs robustly associated with age at first birth, and age at first birth and breast cancer risk Table 14 Association between SNPs robustly associated with age at last birth, and age at last birth and breast cancer risk. Table 15 Association between SNPs robustly associated with number of births, and number of births and breast cancer risk.

Additional file 3: Supplementary Figures. Fig. S1 Multivariable mendelian randomization using MR Egger assessing the effects of ever parous status on overall, ER positive and ER negative breast cancer risk. Fig. S2 Multivariable mendelian randomization using MR Egger assessing the effects of age at first birth on overall, ER positive and ER negative breast cancer risk. Fig. S3 Multivariable mendelian randomization using MR Egger assessing the effects of age at last live births on overall, ER positive and ER negative breast cancer risk. Fig. S4 Multivariable mendelian randomization using MR Egger assessing the effects of number of births on overall, ER positive and ER negative breast cancer risk. Fig. S5 Univariable and multivariable mendelian randomization assessing the effects of age at first birth, on HER2 enriched and triple negative breast cancer risk.

## Data Availability

The availability of all data analysed in this study has been referenced throughout the manuscript and supplementary materials. GWAS summary statistics for breast cancer risk outcomes were obtained from [https://bcac.ccge.medschl.cam.ac.uk/bcacdata/](https:/bcac.ccge.medschl.cam.ac.uk/bcacdata)

## Abbreviations

ER: Estrogen receptor
OR: Odds ratio
MR: Mendelian randomization
MVMR: Multivariable Mendelian randomization
MRC: Medical Research Council
IEU: Integrative Epidemiology Unit
GWAS: Genome-wide association study
BCAC: Breast Cancer Association Consortium
HER2: Human epidermal growth factor receptor 2
LDSC: Linkage disequilibrium score regression
STROBE-MR: Strengthening the Reporting of Observational Studies in Epidemiology using Mendelian Randomization
UVMR: Univariable Mendelian randomization
IVW: Inverse variance weighted
SD: Standard deviation
MR-PRESSO: Mendelian randomisation Pleiotropy RESidual Sum and Outlier
SNP: Single nucleotide polymorphism
