# Supplementary material for: Assessing the causal and independent impact of parity-related reproductive factors on risk of breast cancer subtypes
Source: BMC Med. 2025 Oct 1;23:530. doi: 10.1186/s12916-025-04375-6 (PMC12487161; doi:10.1186/s12916-025-04375-6)
Supplement: Supplementary file 1 — Additional file 1. Supplementary methods and results, and MR STROBE checklist. [file 12916_2025_4375_MOESM1_ESM.docx]

## Methods

### UK Biobank variables

#### Reproductive factors

To identify the number of live births, women were asked "How many children have you given birth to? (Please include live births only)". A binary measure of parous status, coded as “0” or “1”, where women who had or had not given birth, was derived from the measure of number of live births. Women who indicated that they had given birth to one child were asked "How old were you when you had your child?". Women who indicated that they had given birth to more than one child were asked "How old were you when you had your FIRST child?" and "How old were you when you had your LAST child?". To derive age at first live birth and age at last live birth, responses from primiparous and multiparous women were combined.

#### Menstrual traits

Age at menarche was derived from the question: "How old were you when your periods started?". Age at menopause was derived from the question "How old were you when your periods stopped?", this did not include women who had a hysterectomy/were not sure whether they had gone through menopause.

#### Adiposity measures

We explored adiposity in childhood using the comparative body size measure obtained from the baseline questionnaire in UK Biobank. Participants were asked, “When you were 10 years old, compared to average would you describe yourself as:", and were given the options: “Thinner”, “Plumper” and “About average”. We investigated adiposity in adulthood using body size based on BMI. BMI was derived from height and weight measured during the initial UK Biobank Assessment Centre visit. The categorical body size measure was composed on three groups based on the same proportions as the childhood body size variable.

A genetic score of the childhood body size measure has been validated as a predictor of childhood adiposity by previous studies in 3 European cohorts; Trøndelag Health Study, [40] The Young Finn Study, [41] and the Avon Longitudinal Study of Parents and Children. [42] In addition, the polygenic score for childhood body size in UK Biobank was more correlated with childhood obesity in an independent sample compared to the adulthood body mass index (BMI) genome-wide association studies (GWAS). [43] Finally genetic risk scores for childhood body size are more strongly associated with fat mass compared to lean mass. [44]

### Breast Cancer Association Consortium

#### Overall breast cancer

The overall breast cancer risk GWAS summary statistics were obtained from the Breast Cancer Association Consortium (BCAC) which used iCOGS, OncoArray and other GWAS data for 133,384 breast cancer cases and 113,789 controls of European ancestry from 82 studies. [45]

#### Estrogen receptor status

GWAS summary statistics for breast cancer risk stratified by ER status were obtained from BCAC. [46] The data contained 69,501 ER positive cases, 21,468 ER negative cases and 105,974 controls of European ancestry.

#### Estrogen receptor negative subtypes

We additionally investigated the birth-related reproductive factors, which showed an effect on risk of ER positive or negative breast cancer, in relation to intrinsic breast cancer subtypes that are characterised by being hormone-receptor positive or negative. GWAS summary statistics used in this study were for risk of two breast cancer subtypes; human epidermal growth factor receptor 2 (HER2) enriched (718 cases) and triple negative (2,006 cases), were obtained from the BCAC. The data involved individuals of European ancestry and contained 20,815 controls. [45]

### GWAS

BOLT-LMM was used to conduct the analysis in the GWAS pipeline, [37] which accounts for population stratification and relatedness using linear mixed modelling. Genotyping chip and age were included as covariates. Genome-wide significant single nucleotide polymorphisms (SNPs) were selected at p <5×10^−8^ and were clumped to ensure independence at linkage disequilibrium (LD) r^2^ < 0.001 and a distance of 10 000 kb using the TwoSampleMR package.^(9)^ Each GWAS was performed using the Medical Research Council (MRC) Integrative Epidemiology Unit (IEU) UK Biobank GWAS pipeline. [38,39]

### Genetic correlation

Genetic correlation was performed using the linkage disequilibrium score regression (LDSC). The regressions were performed using pre-computed LD scores for each SNP calculated based on individuals of European ancestry from 1000 Genomes European data. [47] These LD scores were filtered to HapMap3 SNPs as these are well-imputed in most studies. [49] SNPs found on chromosome 6 in the region 26MB to 34MB were excluded. GWAS summary statistics were converted for LDSC regression using the munge_sumstats.py command from the command line tool “ldsc”, and LDSC was performed using the ldsc.py command.

### Univariable mendelian randomization

We used the function “mr()” from TwoSampleMR R package. [38] The primary analysis focused on the inverse variance weighted (IVW) MR method. [50] To evaluate evidence of pleiotropy we applied the function “mr()” to perform MR using the weighted mode, weighted median and MR Egger methods, we additionally applied the “pleiotropy_mr()” function to performed the MR Egger intercept test. [38, 51] Furthermore, we applied the “mr_presso” function from the “rondolab/MR-PRESSO” R package. [52] MR and its application have been described extensively elsewhere. [18, 53]

### Multivariable mendelian randomization

We used the “ivw_mvmr()” from the MVMR R package.^(18)^ To evaluate the conditional F statistic, horizontal pleiotropy, and MVMR estimation using Q-statistic minimisation we applied the ‘strength_mvmr()’, ‘pleiotropy_mvmr()’, and ‘qhet_mvmr()’ functions from the “MVMR” R package. [54] We calculated the phenotypic correlation between exposures in using the the MVMR models using Pearson’s correlation coefficient which was required for applying the ‘strength_mvmr()’,  ‘pleiotropy_mvmr()’ and ‘qhet_mvmr()’ functions from the “MVMR” R package. [54]

Additionally we applied the “mr_mvegger” function from the “MendelianRandomization” R package to evaluate evidence of horizontal pleiotropy, and the genetic associations with the first exposure were set to positive. [55]

MVMR and its application have been described extensively elsewhere. [25,26]

## Results

### Estrogen receptor negative subtypes

The F statistic for age at first birth in the univariable analysis was 39.18. However, in the multivariable analysis the F statistic was reduced, falling below 10 with adjustment for at least one of the factors but above 4 allowing use to perform MVMR estimation using Q-statistic minimisation (**Additional File 2: Table 2**). There was evidence of heterogeneity in the MVMR analysis of age at first birth on triple negative breast cancer, but limited evidence on HER2 enriched breast cancer (**Additional File 2: Table 10**).

*MR Egger*

We performed UVMR and MVMR using the MR Egger method of age at first birth on HER2 enriched and triple negative breast cancer. The MR Egger method revealed a similar effect of age at first birth adjusted for HER2 enriched breast cancer however confidence intervals spanned the null. We identified limited evidence when adjusting for ever parous status, conflicting with the inverse effect found in the main analysis. Additionally, the MR Egger method revealed evidence for an effect of age at first birth on HER2 enriched breast cancer adjusted for age at menopause which was not identified in the main analysis. The MR Egger method revealed limited evidence for an effect of age at first birth on triple negative breast cancer (**Additional File 2: Table 11**).

### Evaluating Mendelian randomization assumptions

While there was some consistency between the univariable analysis using the IVW method and the additional MR methods (MR Egger, weighted median and weighted mode) there were inconsistencies in the analysis of ever parous status and age at first birth which may suggest pleiotropy in these analyses. While the effect estimates for ever parous status on overall and ER negative breast cancer risk were largely similar to the IVW method across the additional methods, confidence intervals for the MR Egger and weighted mode spanned the null, with the MR Egger estimate being largely attenuated (**Additional File 2: Table 7**). In addition, the effect of age at first birth on ER negative breast cancer risk was similar when using the weighted median and mode methods but not MR Egger, where the estimate was in the positive direction. However, across all additional methods confidence intervals spanned the null (**Additional File 2: Table 7**).

Using the MR-PRESSO method, we identified potential outliers for all relationships assessed, except for the effect of age at last birth on overall and ER positive breast cancer risk, and the effect of age at first birth on HER2 enriched and Triple negative breast cancer risk. Correcting for identified outliers didn’t appear to change the evidence for effects identified in the initial analysis using the IVW method. However, it is worth noting that of the 4 SNPs used as instruments for ever parous status, 2 were identified as outliers in relation to overall and ER positive breast cancer risk and 1 in relation to ER negative, meaning this analysis was based on a limited number of instruments (**Additional File 2: Table 8**)

In the multivariable analysis we identified evidence of heterogeneity with some exceptions for all models except for those that included: age at first birth and age at last birth; and, age at last birth and ever parous status in relation to ER positive breast cancer risk, those that included age at first birth and age at last birth in relation to ER negative breast cancer risk, and in the analysis of age at first birth for all adjustments except childhood body size on HER2 enriched breast cancer risk. (**Additional File 2: Table 10**).

We performed the multivariable analysis using the MR Egger method to assess for pleiotropy and the genetic associations with the first exposure were set to be positive. The estimates were mostly consistent with the main analysis, although in many cases, the confidence intervals spanned the null. However, there were inconsistencies. Of note, we identified some evidence for an effect of ever parous status on ER positive breast cancer risk adjusted for age at first birth (OR: 0.30, CI: 0.09, 0.97) which we do not find strong evidence for in the main analysis. (**Additional File 2: Table 11, Additional File 3: Fig. S1**) In addition, the main analysis identified strong evidence for an effect of age at first birth on overall breast cancer risk adjusted for ever parous status which was not seen using the MR Egger method. (**Additional File 2: Table 11, Additional File 3: Fig. S2**) The MR Egger method in the multivariable analysis revealed an inverse effect of number of births, adjusted for age at first birth, on overall (OR: 0.49, CI: 0.26, 0.91) and ER negative breast cancer risk (OR: 0.42, CI: 0.19, 0.91) which was not identified in the main analysis. (**Additional File 2: Table 11, Additional File 3: Fig. S4**)

Where we identified an effect using the IVW method of age at first birth on HER2 enriched breast cancer risk in the multivariable analysis, wide confidence intervals included the null using the MR Egger method (adjusted for ever parous status: OR: 1.08, CI: 0.07, 15.71, adjusted for number of births: OR: 0.27, CI: 0.05, 1.62). (**Additional File 2: Table 11**) Although we did identify some evidence for an inverse effect of age at first birth on HER2 enriched breast cancer risk adjusting for age at menopause (OR: 0.46, CI: 0.21, 1.00). (**Additional File 2: Table 11**) We additionally identified minimal evidence for an effect of age at first birth on triple negative breast cancer risk in the MVMR analysis using the MR Egger method, similarly to the IVW method. (**Additional File 2: Table 11**)

## STROBE-MR checklist of recommended items to address in reports of Mendelian randomization studies^1^ ^2^

| **Item No.** | **Section** | **Checklist item** | **Check** | **Page No.** | **Relevant text from manuscript** |
| --- | --- | --- | --- | --- | --- |
| 1 | **TITLE and ABSTRACT** | Indicate Mendelian randomization (MR) as the study’s design in the title and/or the abstract if that is a main purpose of the study |  | Main text: page 1-2 | “We applied univariable and multivariable Mendelian randomization (MR)” |
|  | **INTRODUCTION** |  |  |  |  |
| 2 | **Background** | Explain the scientific background and rationale for the reported study. What is the exposure? Is a potential causal relationship between exposure and outcome plausible? Justify why MR is a helpful method to address the study question |  | Main text: page 3-5 | “It is currently unclear how each reproductive event affects risk in isolation since these traits are highly correlated with, and/or causally linked to other reproductive factors as well as age at menarche and menopause, and adiposity measures, all established breast cancer risk factors.” |
| 3 | **Objectives** | State specific objectives clearly, including pre-specified causal hypotheses (if any). State that MR is a method that, under specific assumptions, intends to estimate causal effects |  | Main text: page 5 |  |
|  | **METHODS** |  |  |  |  |
| 4 | **Study design and data sources** | Present key elements of the study design early in the article. Consider including a table listing sources of data for all phases of the study. For each data source contributing to the analysis, describe the following: |  |  |  |
|  | a) | Setting: Describe the study design and the underlying population, if possible. Describe the setting, locations, and relevant dates, including periods of recruitment, exposure, follow-up, and data collection, when available. |  | Main text: page 6 |  |
|  | b) | Participants: Give the eligibility criteria, and the sources and methods of selection of participants. Report the sample size, and whether any power or sample size calculations were carried out prior to the main analysis |  | Main text: page 6 |  |
|  | c) | Describe measurement, quality control and selection of genetic variants |  | Additional File 1: page 1-2 |  |
|  | d) | For each exposure, outcome, and other relevant variables, describe methods of assessment and diagnostic criteria for diseases |  | Additional File 1: page 1-2, main text: page 6-8 |  |
|  | e) | Provide details of ethics committee approval and participant informed consent, if relevant |  | Main text: page 28 |  |
| 5 | **Assumptions** | Explicitly state the three core IV assumptions for the main analysis (relevance, independence and exclusion restriction) as well assumptions for any additional or sensitivity analysis |  | Main text: page 8-10 |  |
| 6 | **Statistical methods: main analysis** | Describe statistical methods and statistics used |  |  |  |
|  | a) | Describe how quantitative variables were handled in the analyses (i.e., scale, units, model) |  | Main text: page 7 |  |
|  | b) | Describe how genetic variants were handled in the analyses and, if applicable, how their weights were selected |  | Additional File 1: page 2 |  |
|  | c) | Describe the MR estimator (e.g. two-stage least squares, Wald ratio) and related statistics. Detail the included covariates and, in case of two-sample MR, whether the same covariate set was used for adjustment in the two samples |  | Main text: page 8-10 | “The primary analysis focused on the inverse variance weighted (IVW) MR method” |
|  | d) | Explain how missing data were addressed | n/a |  |  |
|  | e) | If applicable, indicate how multiple testing was addressed | n/a |  |  |
| 7 | **Assessment of assumptions** | Describe any methods or prior knowledge used to assess the assumptions or justify their validity |  | Main text: page 9-11 |  |
| 8 | **Sensitivity analyses and additional analyses** | Describe any sensitivity analyses or additional analyses performed (e.g. comparison of effect estimates from different approaches, independent replication, bias analytic techniques, validation of instruments, simulations) | n/a |  |  |
| 9 | **Software and pre-registration** |  |  |  |  |
|  | a) | Name statistical software and package(s), including version and settings used |  | Main text: page 8-10, Additional File 1: page 2,3 |  |
|  | b) | State whether the study protocol and details were pre-registered (as well as when and where) | n/a |  |  |
|  | **RESULTS** |  |  |  |  |
| 10 | **Descriptive data** |  |  |  |  |
|  | a) | Report the numbers of individuals at each stage of included studies and reasons for exclusion. Consider use of a flow diagram |  | Table 2 |  |
|  | b) | Report summary statistics for phenotypic exposure(s), outcome(s), and other relevant variables (e.g. means, SDs, proportions) |  | Table 2 |  |
|  | c) | If the data sources include meta-analyses of previous studies, provide the assessments of heterogeneity across these studies | n/a |  |  |
|  | d) | For two-sample MR:  i.  Provide justification of the similarity of the genetic variant-exposure associations between the exposure and outcome samples  ii.  Provide information on the number of individuals who overlap between the exposure and outcome studies | No overlap | Main text: page 7-8 |  |
| 11 | **Main results** |  |  |  |  |
|  | a) | Report the associations between genetic variant and exposure, and between genetic variant and outcome, preferably on an interpretable scale |  | Additional File 2: Table 15-18 |  |
|  | b) | Report MR estimates of the relationship between exposure and outcome, and the measures of uncertainty from the MR analysis, on an interpretable scale, such as odds ratio or relative risk per SD difference |  | Main text: page 13-15,17,18, Figure 4 |  |
|  | c) | If relevant, consider translating estimates of relative risk into absolute risk for a meaningful time period | n/a |  |  |
|  | d) | Consider plots to visualize results (e.g. forest plot, scatterplot of associations between genetic variants and outcome versus between genetic variants and exposure) |  | Figure 4 |  |
| 12 | **Assessment of assumptions** |  |  |  |  |
|  | a) | Report the assessment of the validity of the assumptions |  | Main text: page 19-20 |  |
|  | b) | Report any additional statistics (e.g., assessments of heterogeneity across genetic variants, such as *I^2^*, Q statistic or E-value) |  | Additional File 2: table 10 |  |
| 13 | **Sensitivity analyses and additional analyses** |  |  |  |  |
|  | a) | Report any sensitivity analyses to assess the robustness of the main results to violations of the assumptions |  | Main text: page 19-20 |  |
|  | b) | Report results from other sensitivity analyses or additional analyses | n/a |  |  |
|  | c) | Report any assessment of direction of causal relationship (e.g., bidirectional MR) | n/a |  |  |
|  | d) | When relevant, report and compare with estimates from non-MR analyses | n/a |  |  |
|  | e) | Consider additional plots to visualize results (e.g., leave-one-out analyses) | n/a |  |  |
|  | **DISCUSSION** |  |  |  |  |
| 14 | **Key results** | Summarize key results with reference to study objectives |  | Main text: page 20-21 |  |
| 15 | **Limitations** | Discuss limitations of the study, taking into account the validity of the IV assumptions, other sources of potential bias, and imprecision. Discuss both direction and magnitude of any potential bias and any efforts to address them |  | Main text: page 23-25 |  |
| 16 | **Interpretation** |  |  |  |  |
|  | a) | Meaning: Give a cautious overall interpretation of results in the context of their limitations and in comparison with other studies |  | Main text: page 20-26 |  |
|  | b) | Mechanism: Discuss underlying biological mechanisms that could drive a potential causal relationship between the investigated exposure and the outcome, and whether the gene-environment equivalence assumption is reasonable. Use causal language carefully, clarifying that IV estimates may provide causal effects only under certain assumptions |  | Main text: page 21 |  |
|  | c) | Clinical relevance: Discuss whether the results have clinical or public policy relevance, and to what extent they inform effect sizes of possible interventions | n/a |  |  |
| 17 | **Generalizability** | Discuss the generalizability of the study results (a) to other populations, (b) across other exposure periods/timings, and (c) across other levels of exposure |  | Main text: page 24-25 |  |
|  | **OTHER INFORMATION** |  |  |  |  |
| 18 | **Funding** | Describe sources of funding and the role of funders in the present study and, if applicable, sources of funding for the databases and original study or studies on which the present study is based |  | Main text: page 27 |  |
| 19 | **Data and data sharing** | Provide the data used to perform all analyses or report where and how the data can be accessed, and reference these sources in the article. Provide the statistical code needed to reproduce the results in the article, or report whether the code is publicly accessible and if so, where |  | Main text: page 28 |  |
| 20 | **Conflicts of Interest** | All authors should declare all potential conflicts of interest |  | Main text: page 28 |  |

This checklist is copyrighted by the Equator Network under the Creative Commons Attribution 3.0 Unported (CC BY 3.0) license.
