# Supplementary material for: Assessing the causal and independent impact of parity-related reproductive factors on risk of breast cancer subtypes
Source: BMC Med. 2025 Oct 1;23:530. doi: 10.1186/s12916-025-04375-6 (PMC12487161; doi:10.1186/s12916-025-04375-6)
Supplement: Supplementary file 3 — Additional file 3: Supplementary Figures. Fig. S1 Multivariable mendelian randomization using MR Egger assessing the effects of ever parous status on overall, ER positive and ER negative breast cancer risk. Fig. S2 Multivariable mendelian randomization using MR Egger assessing the effects of age at first birth on overall, ER positive and ER negative breast cancer risk. Fig. S3 Multivariable mendelian randomization using MR Egger assessing the effects of age at last live births on overall, ER positive and ER negative breast cancer risk. Fig. S4 Multivariable mendelian randomization using MR Egger assessing the effects of number of births on overall, ER positive and ER negative breast cancer risk. Fig. S5 Univariable and multivariable mendelian randomization assessing the effects of age at first birth, on HER2 enriched and triple negative breast cancer risk. [file 12916_2025_4375_MOESM3_ESM.docx]

**Figure S1** Univariable and multivariable mendelian randomization assessing the effects of age at first birth, on HER2 enriched and triple negative breast cancer risk.

No adjustment indicated findings from the univariable analysis.


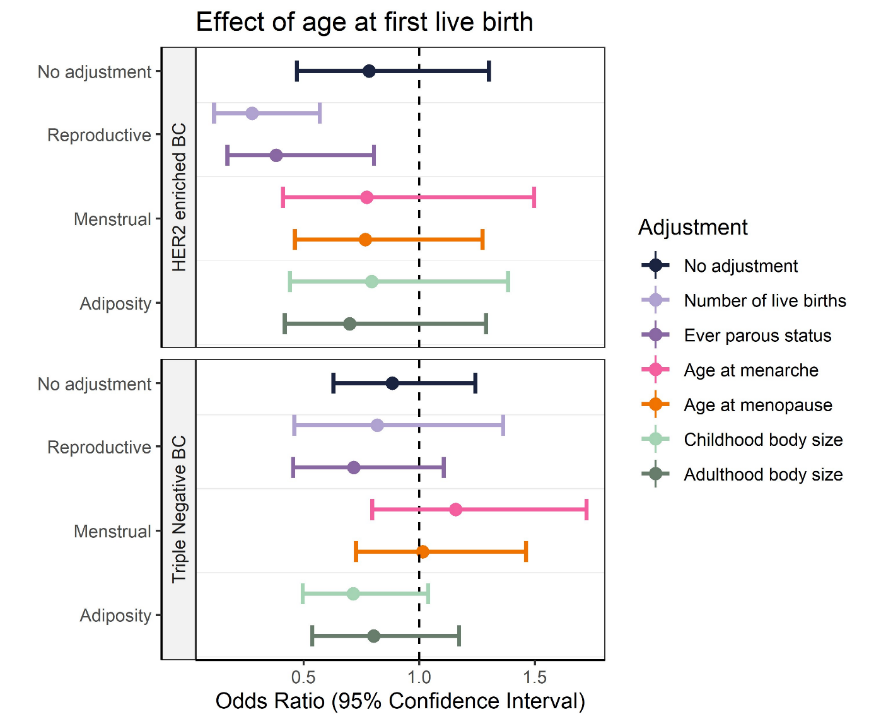


**Figure S2** Multivariable mendelian randomization using MR Egger assessing the effects of number of births on overall, ER positive and ER negative breast cancer risk.


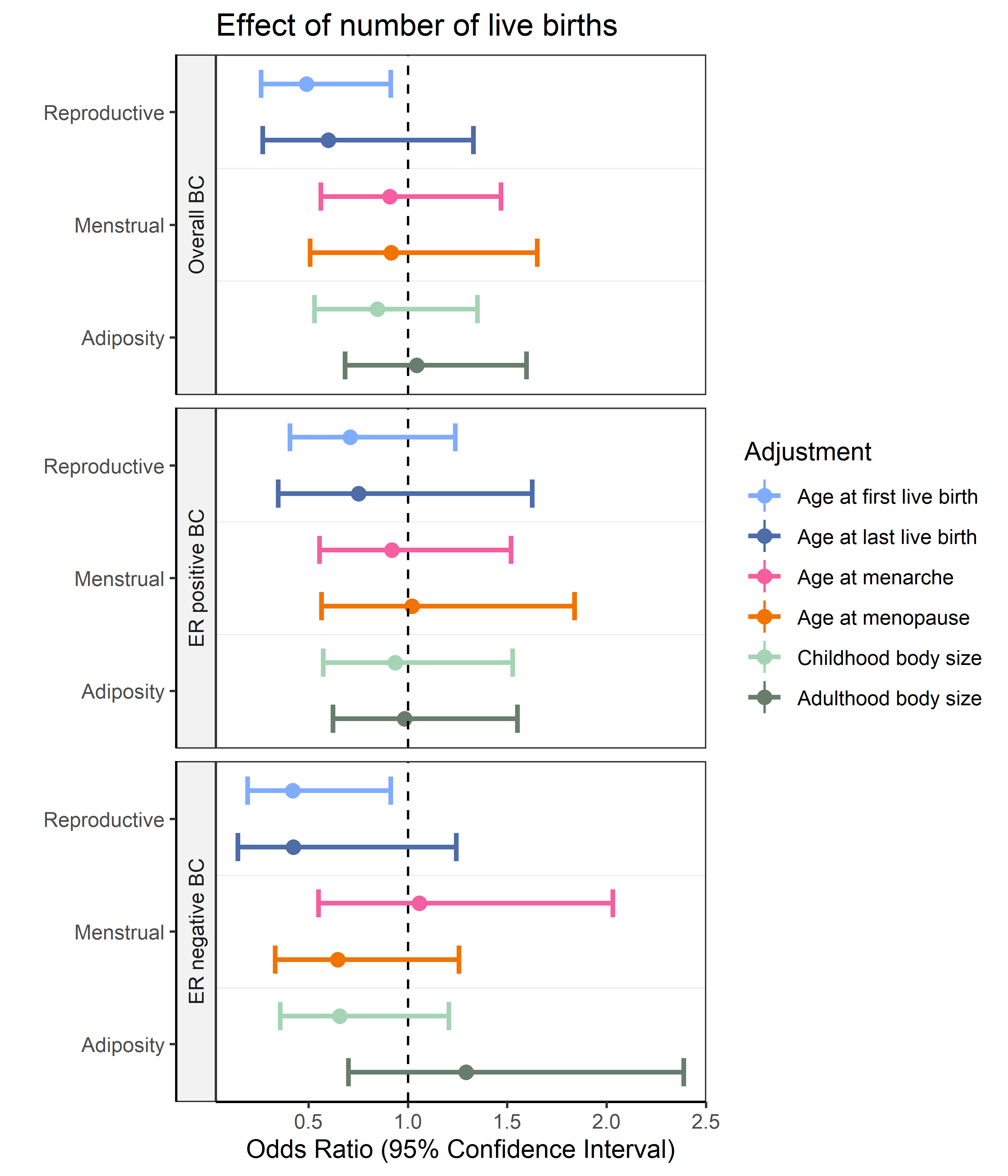


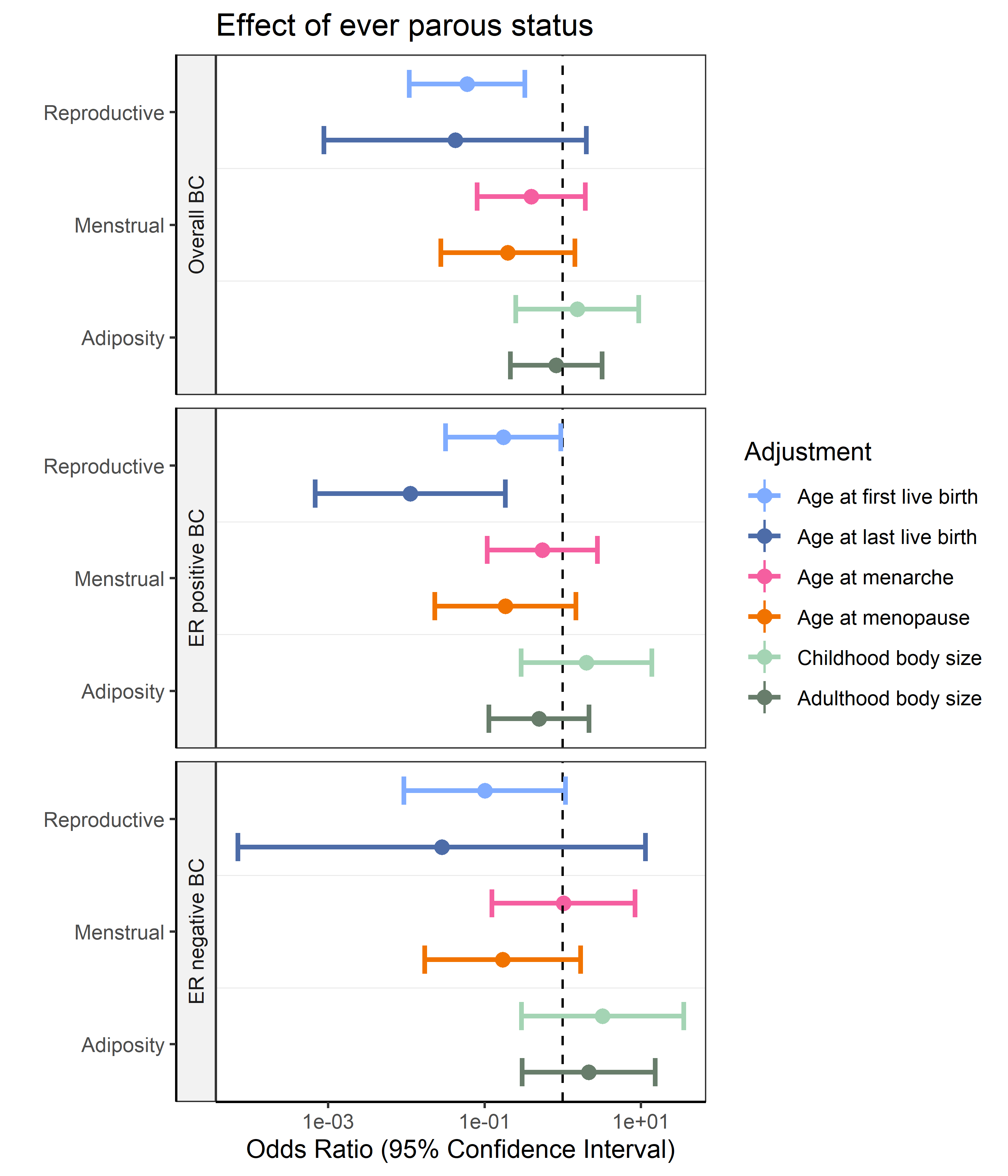


**Figure S3** Multivariable mendelian randomization using MR Egger assessing the effects of ever parous status on overall, ER positive and ER negative breast cancer risk.

**Figure S4** Multivariable mendelian randomization using MR Egger assessing the effects of age at first birth on overall, ER positive and ER negative breast cancer risk.


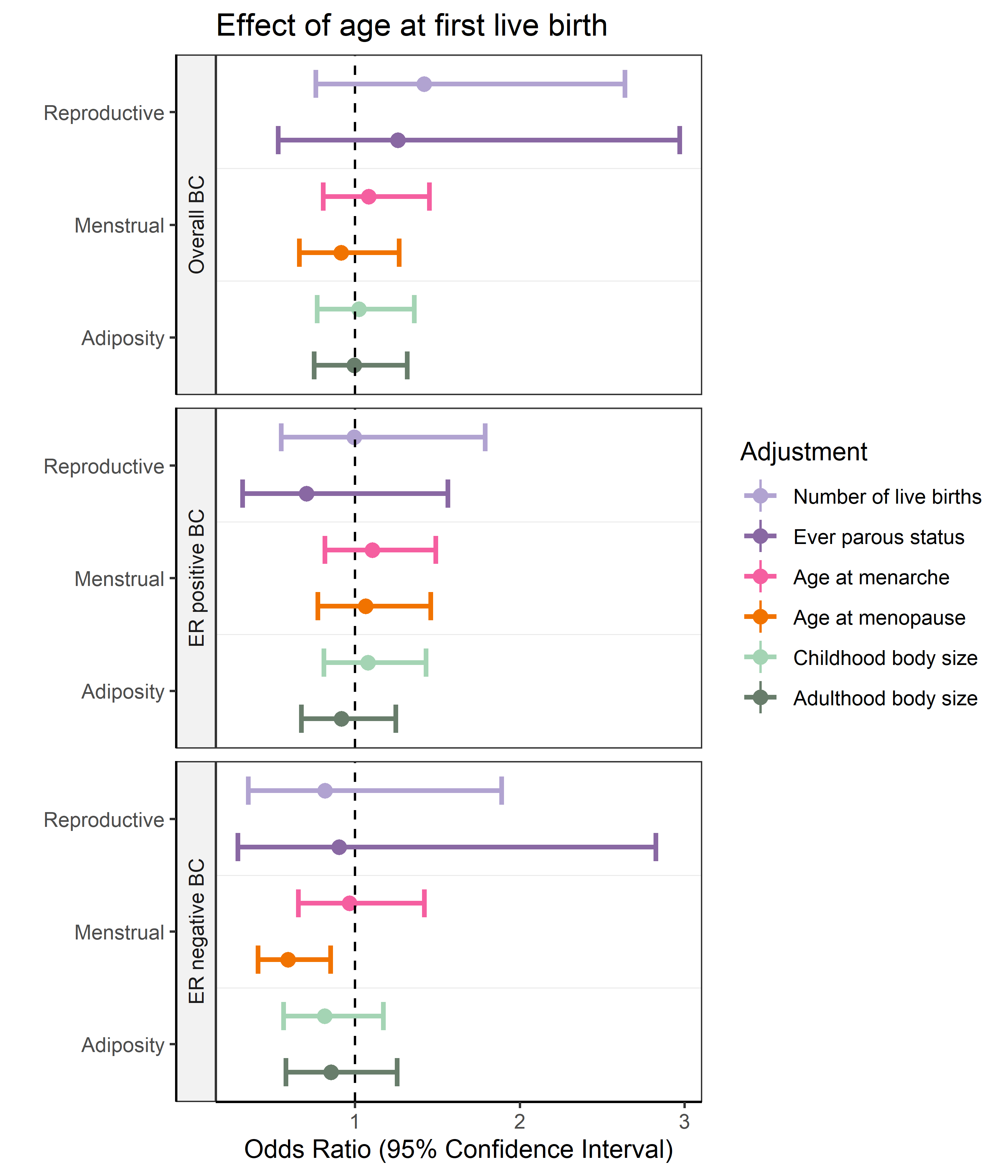


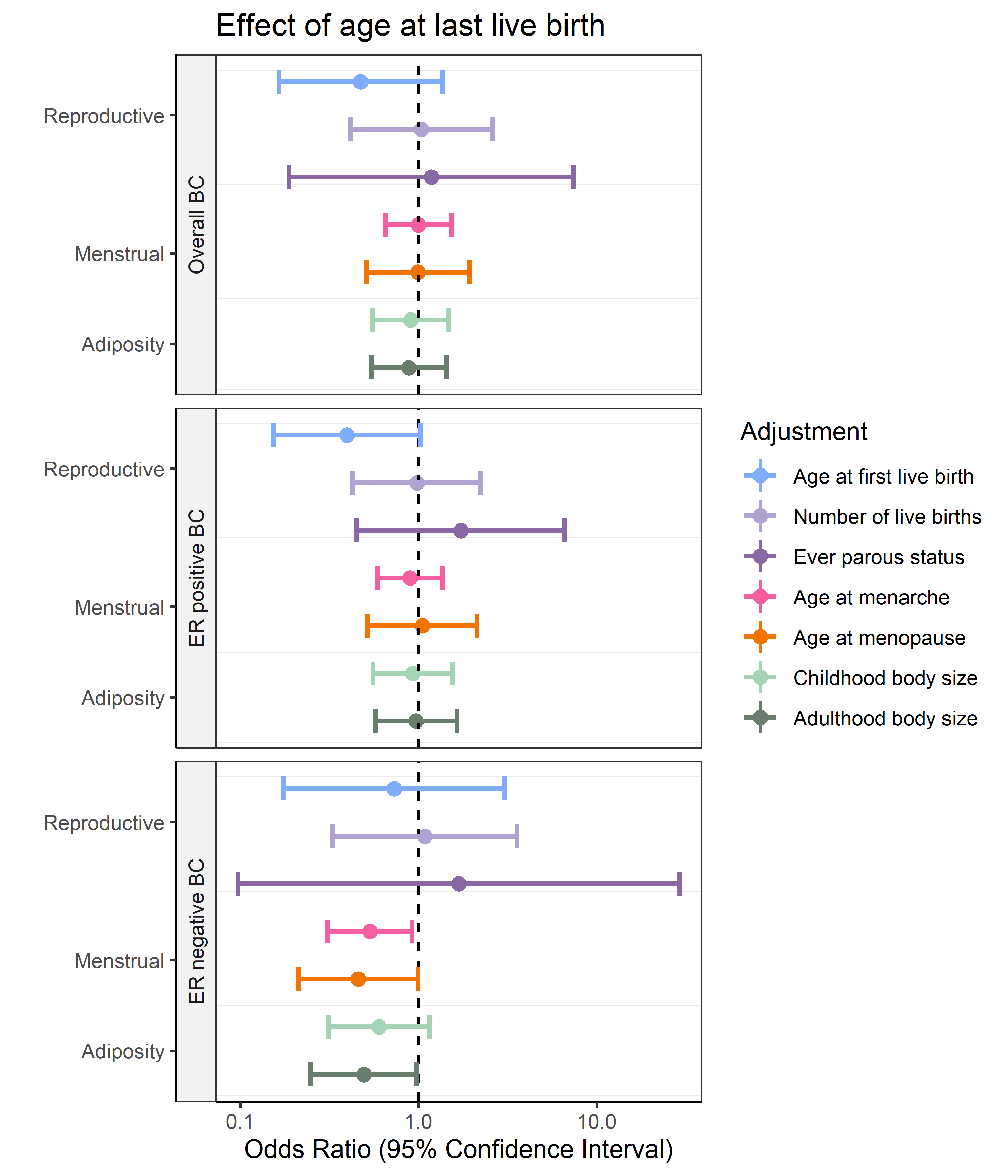


**Figure S5** Multivariable mendelian randomization using MR Egger assessing the effects of age at last live births on overall, ER positive and ER negative breast cancer risk. `
